# The functional brain networks that underlie visual working memory in the first two years of life

**DOI:** 10.1016/j.neuroimage.2020.116971

**Published:** 2020-10-01

**Authors:** Lourdes Delgado Reyes, Sobanawartiny Wijeakumar, Vincent A. Magnotta, Samuel H. Forbes, John P. Spencer

**Affiliations:** a School of Psychology, University of East Anglia, UK; b Department of Psychology, University of Pennsylvania, USA; c School of Psychology, University of Nottingham, UK; d Department of Radiology, University of Iowa, USA

## Abstract

Visual working memory (VWM) is a central cognitive system used to compare views of the world and detect changes in the local environment. This system undergoes dramatic development in the first two years; however, we know relatively little about the functional organization of VWM at the level of the brain. Here, we used image-based functional near-infrared spectroscopy (fNIRS) to test four hypotheses about the spatial organization of the VWM network in early development. Four-month-olds, 1-year-olds, and 2-year-olds completed a VWM task while we recorded neural activity from 19 cortical regions-of-interest identified from a meta-analysis of the adult fMRI literature on VWM. Results showed significant task-specific functional activation near 6 of 19 ROIs, revealing spatial consistency in the brain regions activated in our study and brain regions identified to be part of the VWM network in adult fMRI studies. Working memory related activation was centered on bilateral anterior intraparietal sulcus (aIPS), left temporoparietal junction (TPJ), and left ventral occipital complex (VOC), while visual exploratory measures were associated with activation in right dorsolateral prefrontal cortex, left TPJ, and bilateral IPS. Results show that a distributed brain network underlies functional changes in VWM in infancy, revealing new insights into the neural mechanisms that support infants’ improved ability to remember visual information and to detect changes in an on-going visual stream.

## Highlights

• A distributed brain network underlies functional changes in VWM in infancy and toddlerhood.
• This network shows robust engagement of similar brain regions identified in fMRI studies with adults as early as four months.
• Working memory related activation was centered on bilateral anterior intraparietal sulcus, left temporoparietal junction, and left ventral occipital complex
• Visual exploratory measures were associated with activation in right dorsolateral prefrontal cortex, bilateral anterior intraparietal sulcus, and left temporoparietal junction.

## 1 Introduction

Visual working memory (VWM) is a core cognitive system with a highly limited capacity. This system plays a key role in much of visual cognition, comparing percepts that cannot be simultaneously foveated and identifying changes in the world when they occur (Luck and Vogel, 1997; Vogel et al., 2001). VWM deficits have been observed in clinical populations, including children diagnosed with attention-deficit/hyperactivity disorder and autism (Steele et al., 2007), as well as children born preterm (Vicari et al., 2004). Moreover, individual differences in visual cognition in infancy are predictive of schooling outcomes up to 11 years later (Rose et al., 2012). Given these influences, understanding the early development of VWM has broad implications and may be critical to intervention efforts with at-risk children. Neural measures could usefully contribute to this goal providing biomarkers for risk (Bosl et al., 2018; Tierney et al., 2012) as well as novel information about the mechanisms that underlie the emergence of VWM in early development.

What do we know about the early development of VWM networks in the brain? Several studies have looked at this question by examining correlations between changes in brain structure and infants’^1^ performance in either concurrent or later WM tasks. Short et al. (2013) reported higher fractional anisotropy scores and lower radial diffusivity scores in white matter tracts connecting brain regions thought to support WM in infants who performed better on a visuo-spatial working memory task (for related findings using resting-state fMRI, see Alcauter et al., 2015). Although compelling, such studies provide only an indirect view onto how the brain implements VWM in early development because brain function is not assessed (for discussion, see Cusack et al., 2017; Gilmore et al., 2018).

Other approaches measure brain function directly using task-based neuroimaging with infants. For instance, several studies have measured EEG power and coherence from the scalp as infants perform visual cognitive tasks. Cuevas, Bell, Marcovitch and Calkins (2012) reported that changes in frontal coherence and power predicted improvements in VWM performance at 10 months of age, but not earlier in development. Moreover, a longitudinal study showed that task-specific increases in EEG power become more localized over development which may reflect increased neural efficiency (Bell and Wolfe, 2007).

EEG has relatively poor spatial localization so it is difficult to align such findings with what is known about VWM networks later in life. For instance, Kwon et al. (2002) used fMRI to study VWM in 7 to 22-year-olds. These researchers found WM-related increases in brain activity over age within a fronto-parietal network that included left and right dorsolateral prefrontal cortex (DLPFC), left posterior ventrolateral prefrontal cortex (VLPFC), and left and right posterior parietal cortex (PPC). Interestingly, no areas showed a WM-related decrease in activation over development. Similarly, Geier et al. (2008) found evidence that task-specific WM networks were engaged by 8 years of age, including frontal eye fields (FEF) for shifts of attention, as well as left superior parietal lobule (SPL) and right superior frontal gyrus (SFG) for maintenance of items in VWM. They also found that intraparietal lobule (IPL) and middle frontal gyrus (MFG) contributed to maintenance functions in childhood when the VWM task was difficult (at delays as long as 10 ​s). Generally, WM-related activation showed increases over development; however, inferior frontal gyrus (IFG) showed increases in activation from childhood to adolescence with a decline into adulthood suggesting an improvement in neural efficiency (for related results, see Scherf et al., 2006).

Critically, few studies have used fMRI in early development. The challenges here are numerous, including motion of infants in the scanner and the difficulty of getting infants to engage in a task (see Cusack et al., 2017). A recent study looked at visual cognition in infancy, reporting adult-like spatial organization for faces and scenes in visual cortex (Deen et al., 2017). This work is at the forefront of efforts with fMRI in infants; however, only 9 of 17 infants were included in analysis due to motion artifact. Moreover, this study did not engage infants in a task providing only limited information about functional brain organization in early development (see Gilmore et al., 2018).

An alternative to fMRI is fNIRS. fNIRS enables task-based neuroimaging in infancy but with better spatial localization as compared to EEG. For instance, Wilcox and colleagues (Wilcox et al., 2009; Wilcox et al., 2005, 2008; Wilcox et al., 2014) used fNIRS in a violation-of-expectation task to examine infants’ ability to detect changes in object features. They found that task-related activation decreased from 5 to 12 months in object-related temporal areas suggesting the refinement of ventral stream cortical networks involved in object processing. It is unclear whether these neural changes are indicative of changes in VWM per se as the violation-of-expectation paradigm taps multiple visual cognitive processes (see Schöner and Thelen, 2006). More recent work using a change detection task with 3- and 4-year-olds found increases in left parietal and left frontal activation as the VWM load was increased from 1 to 3 items, as well as an increase in parietal activation from 3 to 4 years (Buss et al., 2014).

Here, we build on this fNIRS work, using an innovative image reconstruction approach (Ferradal et al., 2014; Wijeakumar et al., 2017a) to examine, for the first time, localized task-specific activation of the VWM network in infants 0–2 years of age. This allowed us to directly test 4 hypotheses put forth in the extant literature about the localization of the VWM network in early development:

(1) ***The VWM network in infancy is not localized in fronto-parietal cortex; rather, it is mediated by the medial temporal lobe*** (Káldy and Sigala, 2004). This is consistent with data showing that lower hippocampal volumes in neonatal scans were related to poorer WM performance at 2 years (Beauchamp et al., 2008).
(2) ***The VWM network is mediated by the posterior cortex in infancy with little frontal engagement***. Scherf et al. (2006) found caudate and insula activation in childhood along with a core parietal network, but DLPFC, supplementary eye fields (SEF), and FEF activation were only evident in adolescence and adulthood. Similarly, Klingberg et al. (2002) found an increase in superior frontal sulcus activation from 9 to 18 years, and Kwon et al. (2002) found an increase in DLPFC and VLPFC activation from 7 to 22 years. More recently, Buss et al. (2014) found an increase in frontal activation from 3 to 4 years in a VWM task. It is unknown if the frontal cortex is engaged very early in development.
(3) ***The VWM network is lateralized***. Thomason, Race, Burrows, Whitfield-Gabrieli, Glover, and Gabrieli (2009) reported a right-lateralized VWM network and a left-lateralized verbal WM network in 7- to 12-year-old children. Kwon et al. (2002) reported a right-lateralized visual attention network that spans DLPFC and parietal cortex as well as a left-lateralized network including VLPFC involved in WM-related rehearsal in a study of 7- to 22-year-olds. To date, the laterality of the VWM network in early development has not been examined.
(4) ***The VWM network shows an adult-like cortical spatial organization in infancy***. Deen et al. (2017) reported an adult-like functional spatial organization in cortex in response to visual categories by 4–6 months with subsequent refinement. They suggest that the spatial localization of visual cognitive functions in infancy might be similar to the functional localization revealed in studies with adults.

**Fig. 1:**
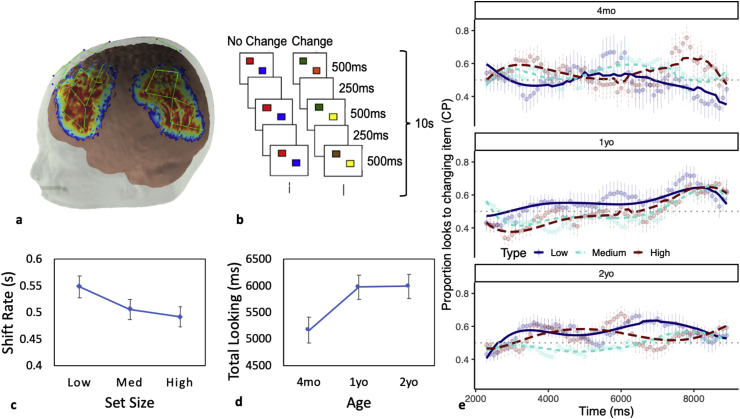
Experimental details and behavioral results. **(a)** Probe geometry laid over the sensitivity profile from an age-matched anatomical template. The figure depicts the regions of the brain we recorded from. Sources are marked with red circles; detectors are marked with blue circles. Channels are shown in green. Figure was created using AtlasviewerGUI (HOMER2, Massachusetts General Hospital/Harvard Medical School, MA, U.S.A.). **(b)** Schematic of a trial of the modified preferential looking task. The stimuli consisted of two side-by-side flickering displays composed of an array of colored squares, one side contained the change display and the other contained the no-change display. Each display contained two, four, or six colored squares. The squares simultaneously appeared for 500 ​ms and disappeared for 250 ​ms during the 10s trials. For the no-change display, the colors of the squares remained constant throughout the length of the trial. For the change display, one of the squares changed color after each delay. **(c)** Shift rate across set size. **(d)** Total looking time across ages. **(e)** Time course model fit to looking data from the task, indicating proportion of looks to the change side (change preference; CP) over time from trial onset. Points and point-ranges indicate means and standard errors of the data; lines indicate model fit. The grey dotted line indicates chance looking at a proportion of 0.5.

To test these hypotheses with image-based fNIRS, we first optimized a probe geometry that would record from regions-of-interest (ROIs) identified from studies of VWM with adults using fMRI (Fig. 1a). In particular, we Identified 21 regions of interest (ROIs) from a meta-analysis of the adult fMRI literature on VWM (see Wijeakumar et al., 2015). We then designed an fNIRS probe that would record from 19 of the 21 ROIs robustly across development (two of the ROIs were too deep to record from using fNIRS; see Wijeakumar et al., 2015).

We used this geometry as 4-month-olds, 1-year-olds, and 2-year-olds completed a preferential looking (PL) task that has been shown to measure changes in VWM in early development (Fig. 1b; Ross-Sheehy et al., 2003). In particular, Ross-Sheehy et al. reported that 4- to 6.5-month-olds preferred a single-item changing display over a single-item non-changing display – a so-called ‘change preference’ – when they were asked to remember the items over a short delay; by contrast, when each display contained two or more items, these infants looked equally at both displays. By 10 months, infants showed a robust change preference with displays as large as 4 items, suggesting an increase in VWM capacity in the first year. Importantly, 6.5-month-old infants showed a robust change preference when the delay was eliminated, showing that the pattern of results reflects a memory limitation rather than a perceptual or attentional limitation (Kwon et al., 2014; Oakes et al., 2011; Oakes et al., 2006). We made one adjustment to this task based on recent computational modelling work (Perone et al., 2011)—we decreased the trial duration from 20s to 10s to reduce infants’ reliance on long-term memory processes and ensure they used VWM to solve the task.

In summary, our goal in the present study was to measure localized, task-specific activation of the VWM network in early development, and how this network changes in the first two years of life. This allowed us to test 4 competing hypotheses about the brain systems underlying this cognitive system. We hope to shed light on the neural mechanisms underlying performance in the preferential looking task, and what changes in the brain to support infants’ improving ability to detect changes in a visual stream. Ultimately, this information and the innovative methods used here may help identify neural biomarkers for children at-risk for VWM deficits early in life.

## 2 Methods

### 2.1 Participants

Seventy-seven infants participated in the study. Children were recruited from a child registry maintained by the Department of Psychology at the University of Iowa. Parents were sent an informational letter inviting them to participate and were later contacted via phone or email. All children had normal or corrected to normal vision. The study was approved by the institutional review board (IRB) at the University of Iowa in compliance with ethical regulations and standards. All participants provided written informed consent. Data from 20 participants were excluded from final analysis due to poor digitizations (4) or poor quality fNIRS data (under/over-saturated signals; 16). The remaining participants were grouped into three age groups: 4-month-olds (*N* ​= ​16, *M* ​= ​17.3 weeks, *SD* ​= ​1.8 weeks, 7 girls), 1-year-olds (*N* ​= ​19, *M* ​= ​64.3 weeks, *SD* ​= ​7.2 weeks, 10 girls), and 2-year-olds (*N* ​= ​22, *M =* 114.0 weeks, *SD* ​= ​4.7 weeks, 12 girls).

Forty-four additional participants were recruited to participate in the study but were excluded for the following reasons: a later discovered excluding medical diagnosis (1), behavior not codable (i.e., excessive movement or standing up during task; 6), pulled the cap off during data collection (6), did not complete enough trials (10), or fussiness during the session (21).

### 2.2 Stimulus and apparatus

We used the Preferential Looking task developed by Ross-Sheehy et al. (2003). A 46-inch LCD television that was connected to a PC running Adobe Director was used to display the stimuli. The stimuli consisted of two side-by-side flickering displays composed of an array of colored squares (Fig. 1c). One side contained the change display and the other contained the no-change display. Each display contained colored squares that measured approximately 5 ​cm (w) by 5 ​cm (h). The set size (number of items in each array) was the same between the two displays and remained constant during the 10s trials. The colors of the squares were selected from a set of nine colors: green, brown, black, violet, cyan, yellow, blue, red and white. The colors on a display were always different from each other but colors could be repeated between the displays (i.e., the same color could appear on both displays).

The squares simultaneously appeared for 500 ​ms and disappeared for 250 ​ms during the 10s trials. For the no-change display, the colors of the squares remained constant throughout the length of the trial. For the change display, one of the squares changed color after each delay. The changing square was randomly selected, and its color was derived from the set of colors not currently present in that display.

### 2.3 Procedure and design

During the task, infants were seated on the parent’s lap or in a high chair in front of the LCD television. An attention getter in the form of a flashing red light paired with an audible tone played at the beginning of every trial to ensure that infants were looking at the center of the screen. A trained observer initiated the trials when the infant was looking at the screen. On a set size 2 (SS2) trial, an infant would see two squares both on the left and right display. There was a 5s inter trial interval. Note that, in practice, this interval varied because a trial was not initiated until the infant was looking at the display following the attention getter.

The observer was unaware of the side of the changing stimulus on each trial and recorded infants’ look durations online by pressing two designated keys, one for when the infant looked at the left display (4) and another for when the infant looked at the right display (6). No keys were pressed when the infant was not looking at one of the two displays. If the infant did not look at the displays during the first 5s of the trial, the trial was repeated. During periods of inattention or fussiness, we presented brief clips of an entertaining children’s music video. Additional clips of the same show were presented every six trials to maintain the infants’ interest in the task. Parents were instructed to keep their eyes closed or wore occluded glasses that blocked view of the screen to minimize bias and were instructed not to interact with the infants during the experiment.

Each infant was presented with a maximum of 36 trials (or the total number of trials the infant would tolerate before they became bored with the task). To conform with previous studies (Oakes et al., 2011, 2006; Ross-Sheehy et al., 2003), the set size varied across trials with low, medium, or high loads (1,2,3 items for the 4mo group; 2, 4, 6 items for the older groups). There were twelve trials per set size; six had the changing stream on the left, while the remaining six had the changing stream on the right. The order of these trials was randomized. Each infant received a different order of stimuli.

### 2.4 Behavioral analysis

The time each infant spent looking at each display (left and right) was recorded online across each 10s trial, rendering their total looking time (TL). Switch rate (SR) in seconds was calculated as the number of times the infant switched from one side to the other divided by total looking time in seconds ((# of switches) ​÷ ​(Total Looking Time ​÷ ​1000)). Looking to the change side and non-change side at each point in time in the trial was aggregated into 100 ​ms time bins, calculating the proportion of looks to the target (change side). To allow for the best possible statistical modelling of these time series data, the data was trimmed to start at 2300 ​ms (at which point participants would have seen 3 full presentations) and end at 9000 ​ms (the last second of data is noisy because fewer participants maintained attention for the full 10s trial duration).

### 2.5 fNIRS data acquisition and analysis

fNIRS data were collected at 25Hz using a TechEn CW6 system with 690 ​nm and 830 ​nm wavelengths. Near-infrared light was delivered via 12 fiber optic cables (sources) to the participant’s scalp and detected by 24 fiber optic cables (detectors) spaced into four arrays (see Fig. 1a). Each array contained three sources and six detectors placed over the frontal, temporal and parietal cortex bilaterally. Previous work showed that this cap geometry records from 19 of 21 ROIs identified by a meta-analysis of the adult fMRI literature on VWM, and that these ROIs are within the range of fNIRS sensors when the geometry is scaled by head circumference over development (Wijeakumar et al., 2015). Optodes were fitted within a custom EEG cap that contained grommets to secure the fiber optics to the scalp. Optode positions were recorded in 3-dimensions using a Polhemus Patriot system before the task.

*Pre-Processing of fNIRS data.* The NIRS data were processed on a channel-by-channel basis using HomER2 (Huppert et al., 2009) (www.nmr.mgh.harvard.edu/PMI/resources/homer2). Raw optical signals were first converted to optical density units. Channels with very low optical density (<80 ​dB; dB ​= ​20∗LOG10(*y*), where *y* is the intensity level measured by the CW6 system) were discarded from the analysis. Signal changes with amplitude greater than 0.5au within 1s or with a SD greater than 50 were identified as motion artifacts. A targeted Principal Component Analysis (Yücel et al., 2014) was then applied for motion correction. Trials with remaining motion epochs within 16 ​s after the stimulus onset after correction were removed from the analysis. Data were then band-pass filtered (0.016–0.5 ​Hz) and the concentrations of oxygenated hemoglobin (HbO), deoxygenated hemoglobin (HbR), and total hemoglobin (HbT) were computed using the modified Beer-Lambert Law. A differential path length (DFP) factor of 6 was used for both wavelengths (Strangman et al., 2003). Recordings from source-detector pairs with short distances (<10 ​mm) were used as regressors to remove physiological fluctuations (Saager and Berger, 2008; Zhang et al., 2009). A general linear model was run on each chromophore separately with regressors that captured stimulus timing and duration for the three conditions of interest (low, med, high) as well as nuisance regressors. Each regressor was convolved with a canonical gamma function (for details, see HomER2 ‘hmrDeconvHRF_DriftSS’ function; HbO parameters: tau ​= ​0.1, sigma ​= ​3.0, T ​= ​10.0; HbR parameters: tau ​= ​1.8, sigma ​= ​3.0, T ​= ​10.0). This resulted in a β estimate for each channel, for each condition for both HbO and HbR per participant.

*Forward Model.* Age-specific atlases (4–6mo, 1yo, and 2yo) from the Neurodevelopmental MRI database were used to estimate a forward head model (Fillmore et al., 2015; Richards et al., 2016; Richards and Xie, 2015). Each atlas was segmented into tissue types (grey matter, white matter, cerebro-spinal fluid and scalp) using 3dSeg from AFNI (Analysis of Functional Neuroimaging; W. Cox, 1996). 3D surface meshes were created from these tissue types using HOMER2 (Wijeakumar et al., 2017a). Digitized scalp landmarks and positions of sources and detectors were projected onto the age-specific atlases and Monte Carlo simulations with 100 million photons were run to create sensitivity profiles for each channel for each participant (Fig. 1a). The head volumes and sensitivity profiles were converted to NIFTI format. Participants’ sensitivity profiles were summed together, thresholded at an optical density value of 0.0001 (see Wijeakumar et al., 2015), and transformed to MNI space to create subject-specific masks. Participant-specific masks from each age were summed together to create age-specific masks. Within each of these age-specific masks, only those voxels that contained data from at least 75% of the participants were taken forward to final analyses. Finally, all thresholded age-specific masks were combined to create an intersection mask.

*Image Reconstruction.* The image reconstruction approach used here is similar to image reconstruction approaches proposed by Ferradal et al. (2014) and Huppert et al. (2017). Note that these approaches have been validated previously by simultaneously recording fNIRS with other imaging modalities (e.g., fMRI; see Wijeakumar et al., 2017a; Huppert et al., 2017). The methods for our image reconstruction approach have been discussed in previous work (Putt et al., 2017; Wijeakumar et al., 2017a, Wijeakumar et al., 2017b; see also Jackson et al., 2019; Putt et al., 2019; Wijeakumar et al., 2019; Wijeakumar et al., 2017a, Wijeakumar et al., 2017b). Briefly, after accommodating for the forward model and beta coefficients from the GLM (see above), the relationship between the hemodynamic response and delta optical density is given by:

$$\left[\begin{array}{c} d\;.\;\varepsilon_{HbO}^{\lambda 1}\;.\;\beta_{HbO}+\;d\;.\;\varepsilon_{HbR}^{\lambda 1}\;.\beta_{HbR}\;\\ d\;.\;\varepsilon_{HbO}^{\lambda 2}\;.\;\beta_{HbO}+\;d\;.\;\varepsilon_{HbR}^{\lambda 2}\;.\;\beta_{HbR} \end{array}\right]=\;\left[\begin{array}{cc} \varepsilon_{HbO}^{\lambda 1}\;.\;\;F^{\lambda 1} & \varepsilon_{HbR}^{\lambda 1}\;.\;\;F^{\lambda 1}\\ \varepsilon_{HbO}^{\lambda 2}\;.\;\;F^{\lambda 2} & \varepsilon_{HbR}^{\lambda 2}\;.\;\;F^{\lambda 2} \end{array}\right]\;.\text{ ​}\left[\begin{array}{c} \text{Δ}HbO_{vox}\\ \text{Δ}HbR_{vox} \end{array}\right]$$

where, *F* is the channel-wise sensitivity volumes from the Monte Carlo simulations. *ΔHbO*_*vox*_ and *ΔHbR*_*vox*_ are voxel-wise relative changes in HbO and HbR concentrations and need to be estimated using an image reconstruction approach. We can re-write this equation as:

Y=L.X

where,

$$\text{Y}=\left[\begin{array}{c} \beta_{dOD}^{\lambda 1}\\ \beta_{dOD}^{\lambda 2} \end{array}\right],\;\text{L}=\left[\begin{array}{cc} \varepsilon_{HbO}^{\lambda 1}\;.\;\;F^{\lambda 1} & \varepsilon_{HbR}^{\lambda 1}\;.\;\;F^{\lambda 1}\\ \varepsilon_{HbR}^{\lambda 2}\;.\;\;F^{\lambda 2} & \varepsilon_{HbR}^{\lambda 2}\;.\;\;F^{\lambda 2} \end{array}\right],\;\text{and}\;\;\text{X}=\left[\begin{array}{c} \text{Δ}HbO_{vox}\\ \text{Δ}HbR_{vox} \end{array}\right]$$

To solve for X, we used Tikhonov regularization and the system in the above equation can be replaced by a ‘regularized’ system given by,

$$X={\left(L^{T\;}L+\;\lambda .I\right)}^{-1}\;L^{T}\;.\;\;Y$$

where *λ* is a regularization parameter that determines the amount of regularization and *I* is the identity operator. Minimizing the cost function and solving for X yields voxel-wise maps of relative changes in concentration for each condition, channel, participant, and chromophore.

### 2.6 Statistical analyses

Visual exploratory measures (shift rate, total looking time) were analyzed using ANOVA with SS (low, medium, high) as a within-subjects factor and Age (4mo, 1yo, 2yo) as a between-subject factor. We report multivariate *F* tests (Wilks’ Lambda) for all ANOVA results because these tests do not require the assumption of sphericity. Change preference scores through time were fit with a binomial hierarchical model estimated with Laplace approximation using the glmmTMB package (Brooks et al., 2017) and eyetrackingR (Dink and Ferguson, 2016) in the statistical package R. The model was fit with quintic orthogonal polynomials of the time term (Mirman, 2014), that is, the data were modelled with time, time squared, up to time to the power 5, but scaled and centered so as to not be correlated with one another. In addition, the model contained fixed effects of Age (4-month-olds, 1-year-olds, 2-year-olds) and SS (low, medium, high). The slope of SS, as well as each of the five time terms was nested as a random effect within participant, along with allowing each participant a random intercept for a maximally-specified model.

fNIRS data were analyzed at the group level using ANOVA on the voxel-wise beta maps. The ANOVA had two within-subjects factors – SS (low, medium, high) and chromophore (HbO, HbR) – and one between-subjects factor – age (4mo, 1yo, 2yo). Only statistically significant main effects and interactions that included chromophore are discussed (i.e., Hb, Age x Hb, SS x Hb, and Age x SS x Hb effects). HbO and HbR are typically anti-correlated in functional neuroimaging studies with HbO ​> ​HbR; thus, by including only effects with a significant difference between chromophores, we ensured that all effects had a good signal to noise ratio with a clear signature of neural activation. The ANOVA was conducted using the 3dMVM function in AFNI. We included the -GES flag to obtain effect size estimates (see Table 1), the -resid flag to model the spatial autocorrelation present in the data (see below), the -wsMVT flag for multivariate testing of all within-subjects effects, and type 2 testing for the sum of squares of the omnibus F-statistics. This analysis was constrained to the portion of the brain covered by the group-level intersection mask (total number of voxels in the mask was 23149 with a voxel size of 2 ​× ​2 ​× ​2 mm^3).

**Table 1:** ANOVA results.

Effect	Cluster	ROI	Hemi	Size	Center of Mass			GES (ηG2)
				(mm^3)	x	y	z	
Chromophore (Hb)	Middle Frontal Gyrus	IFG	R	536	−39	−40	19	0.02
	Inferior Parietal Lobule		L	460	47.9	27.9	54	0.03
	Superior Temporal Gyrus	TPJ	L	380	59	50.2	16	0.04
	Angular Gyrus		L	126	50.7	65	41	0.04
Age Group x Chromophore (Hb)	Middle Frontal Gyrus	DLPFC	R	223	−30	−37	30	0.04
	Angular Gyrus	aIPS	L	197	45.3	63.6	40	0.04
	Middle Temporal Gyrus	VOC	L	147	52.6	56.3	9.6	0.04
Age Group x Set Size x Chromophore (Hb)	Inferior Parietal Lobule	aIPS	R	99	−50	43.4	44	0.03

**Note:** Clusters were localized using the center of mass xyz coordinates and labels were derived from the MNI atlas (Eickhoff-Zilles ​macro ​labels ​from ​N27 in AFNI). The ROI column indicates that a portion of the cluster was overlapping or near a target ROI.

Supplementary linear contrasts were run using the general linear testing approach in 3dMVM. This is like running supplementary ANOVAs but offers the advantage of putting this in the framework of t statistics which indicate directionality (see Chen et al., 2014). We ran a linear contrast of Age by including two contrasts looking at the interaction of chromophore with pairwise ages. The first Age contrast examined the interaction of chromophore (HbO ​> ​HbR) and the two early ages with 4mo ​< ​1yo. The second Age contrast examined the interaction of chromophore and the older ages with 1yo ​< ​2yo. A conjunction of significant effects from these two Age contrasts can be used to examine the presence of linear trends (that is, clusters where 4mo ​< ​1yo AND 1yo ​< ​2yo). Linear effects of SS were examined in a similar manner by looking at pairwise contrasts and then computing the conjunction. In each case, we examined the interaction of chromophore (HbO ​> ​HbR) with SS, comparing SS low ​< ​SS med in the first contrast and comparing SS med ​< ​SS high in the second contrast.

The ANOVA and supplementary linear contrasts were corrected for multiple comparisons (i.e., family-wise errors) using 3dClustSim. Recent papers have raised concerns about inflated false-positive rates using parametric methods like 3dClustSim due to mistaken assumptions about the Gaussian nature of the spatial autocorrelation function (ACF) in neuroimaging data (see Eklund et al., 2016). In response, Cox et al. (2017) proposed a mixed-ACF approach that estimates the empirical ACF with a function that mixes a Gaussian and monoexponential function. The estimated ACF can then be used in 3dClustSim instead of the canonical Gaussian assumption. Cox et al. demonstrated that this approach effectively controls the false-positive rate. In particular, simulations of two large-scale, event-related datasets showed low false-positive rates using the mixed-ACF approach with 3dClustSim with a voxelwise *p* ​= ​0.01 and alpha ​= ​0.05.

**Fig. 2:**
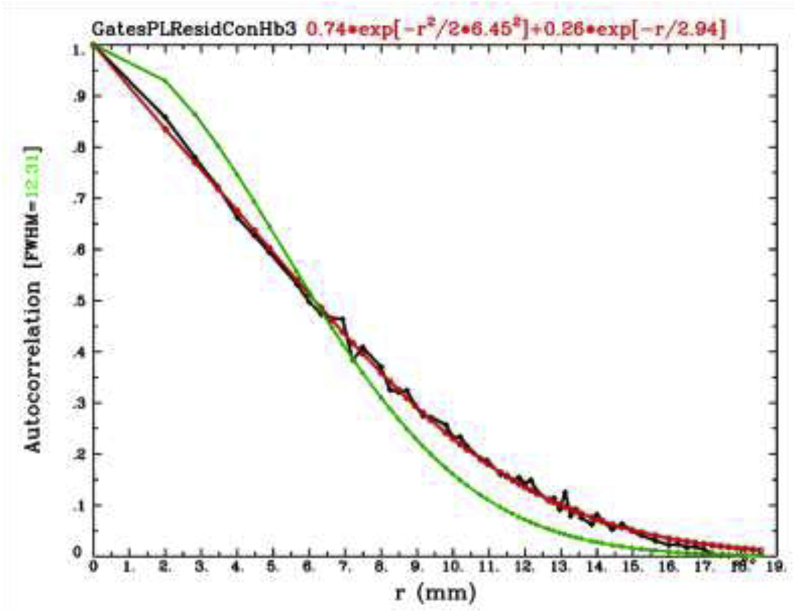
Fit of the mixed ACF model to the empirical ACF in our fNIRS data. Green line depicts the canonical Gaussian ACF assumption, while black line shows the empirically estimated ACF values generated by the program 3dFWHMx. The red line shows the estimated mixed model after fitting parameters described in Cox et al. (2017).

We used this suite of tools to control the family-wise error in our data. In particular, we used the 3dFWHMx function in AFNI to estimate the empirical ACF in our fNIRS data and fit the mixed ACF model to this function. Consistent with fMRI results, our fNIRS data show an undershoot of the Gaussian assumption (green line) at small distances and an overshoot at large distances (see Fig. 2). Critically, the mixed-ACF function provides a good approximation of the empirical ACF. We then used the mixed-ACF parameters (0.7363, 6.4542, 2.9442) in 3dClustSim with a voxelwise *p* ​= ​0.01, alpha ​= ​0.05, and 10,000 iterations. We opted for a voxelwise threshold of *p* ​= ​0.01 because Cox et al. (2017) showed that this criterion value effectively controlled the familywise error rate with two large-scale, event-related datasets with little improvement in the false positive rate when the voxelwise threshold was set to *p* ​= ​0.005. We selected two-sided thresholding with the NN1 option (first-nearest neighbour clustering where above threshold voxels cluster together if faces touch). The cluster size criterion was 98 voxels.

To investigate brain-behavior relationships, we focused solely on clusters with significant chromophore effects in the ANOVA. We considered using a standard correlational approach to examine brain-behavior relationships. Given the presence of clear developmental patterns in both the behavioral and brain data (see results), however, such an approach would have to be run on each age group separately. Moreover, our focus was on brain regions showing a significant chromophore effect (HbO ​> ​HbR), suggesting that we should examine correlations for both chromophores. With three behavioral measures of interest (change preference scores, total looking time, and shift rate), this would result in 144 correlations (8 clusters ∗ 3 age groups ∗ 2 chromophores ∗ 3 behavioral measures). More importantly, the correlation – while asking a basic question about a linear relationship between brain and behavior – fails to model the data fully. The alternative is to model the data from each cluster considering the details of the design and including the behavioral measure as a continuous quantitative predictor. This allowed us to ask a much richer statistical question: if behavior is related to brain activity, how does this relation vary as a function of the factorial structure of the study including Age, SS, and Chromophore as predictors?

We considered two ways to the model the brain-behavior relationships in this context. One option was to use a linear mixed-effect model with Age, SS, Chromophore, and behavioral measure as fixed effects and a random intercept for subject. A second option was to run a simple linear model with Age, SS, Chromophore, and behavioral measure as predictors. We evaluated these approaches with a few clusters. Models were assessed initially using an intercepts-only linear mixed-effects model, that is, a linear model where Beta was predicted by Age, SS, Chromophore and the behavioral variable, while allowing the intercept to vary for each participant. Formal model comparison techniques demonstrated that the random intercept for each participant contributed little to the model fit. Thus, models were simplified to a linear model with Beta predicted by Age, SS, Chromophore and a behavioral variable. In total, we ran 24 models (8 clusters ∗ 3 behavioral measures). We used the omnibus *F* from each model to correct for multiple comparisons using the Benjamini-Hochberg procedure with alpha ​= ​0.05.

Initial exploration of this linear modelling approach indicated that outliers had a strong effect on the models in many cases. Outliers were, therefore, removed from the data using boxplot.stats in R. In particular, points beyond a cut-off equal to the ’hinges’ (approximately the 1st and 3rd quartiles) ±1.5 times the interquartile range were removed, ensuring that that the hinges and whiskers were drawn at points representing actual observations. 12.9% of observations were initially classified as outliers from the overall group dataset; however, we noticed in some clusters that outlier removal was heavily biased toward one age group. Thus, we removed outliers for each age group separately. This resulted in the removal of 10.7% of observations for 4-month-olds (out of 96 total observations), 10.0% for 1-year-olds (out of 114 total observations), and 11.6% for 2-year-olds (out of 132 total observations). In summary, then, fewer observations were removed with this age-specific approach and the model fits were comparable (as evaluated using quantile-quantile plots).

## 3 Results

*Behavioral results*. Looking behaviors were coded on-line by trained observers as in previous studies (see Ross-Sheehy et al., 2003). Visual exploratory measures (shift rate and total looking time) were analyzed using ANOVA with SS (low, medium, high) and Age (4mo, 1yo, 2yo) as factors. There was a significant decrease in shift rate as the set size increased, *Λ* ​= ​0.86, *F*(2,53) ​= ​4.22, *p* ​= ​0.020, *η*_p_^2^ ​= ​0.137, replicating findings from Simmering (2016). As can be seen in Fig. 1c, participants shifted back and forth between displays at a slower rate with higher memory loads as more time was needed to consolidate the items in working memory. No other shift rate effects reached significance. There was also an increase in total looking time with Age, *F*(2,54) ​= ​3.69, *p* ​= ​0.031, *η*_p_^2^ ​= ​0.12, again replicating findings from Simmering (2016). As visual exploratory abilities improved with age, children engaged with the task more, increasing total looking time (see Fig. 1d). No other total looking time effects reached significance.

Looking proportions were modelled with a hierarchical binomial model to examine the effects of change preference, SS, and Age over time (Fig. 1e). The model utilized orthogonal quintic polynomials of the time term to capture the model fit (Mirman, 2014). Fixed effects were tested with a Wald chi-squared test to assess the contribution of each parameter in reducing residual deviance of the model. The results indicate evidence for an interaction effect between the linear, cubic, and quartic time terms and Age, an effect of all five time terms and SS, as well as all 3-way interactions (see supplementary Table S1). Thus, there is some evidence that the time course of looking varies by age, strong evidence that time course of looking to the change side varies by SS, and evidence that the amount by which the time course of looking to the change side varies at each SS differs across age groups.

The model fit to the raw data can be seen in Fig. 1e. Contrasting performance across age groups, it is evident that 4-month-olds’ change preference scores showed considerable fluctuations through time, with above chance looking to the changing side in the medium load condition toward the middle of the trial and above chance looking in the high load condition early and late in the trial. While variability is typical in the performance of this age group, at the group level, 4-month-olds usually show robust change preference scores only in the low load condition (see Ross-Sheehy et al., 2003). One-year-olds, by contrast, showed a robust change preference in the low load condition by 4 ​s and a later emerging change preference in the other conditions by 7 ​s, replicating the above-chance performance of this age group reported by Ross-Sheehy et al. (2003). Two-year-olds showed a similar pattern, although this age group showed above-chance performance in the high load condition by 3–4 ​s suggesting faster detection of the changing side at 2 years.

*fNIRS results*. Table 1 presents the ANOVA results, and Table 2 presents the linear contrast results. Eight clusters showed significant task-specific brain activity in the ANOVA after familywise correction – 4 clusters showed an Hb effect, 3 clusters showed an Age x Hb effect, and 1 cluster showed an Age x SS x Hb effect. In addition, the supplementary Age linear contrasts revealed 5 significant clusters, and the supplementary SS linear contrasts revealed 1 significant cluster. We examine these effects below, first focusing on the Hb, Age x Hb, and Age contrasts. We then examine the SS-related effects (Age x SS x Hb, SS contrasts).

**Table 2:** Linear contrast results.

Contrasts	Cluster	ROI	Hemi	Size	Center of Mass			t-contrasts results
				(mm^3)	x	y	z	
**Age x Hb** 4mo v 1yo	Superior Temporal Gyrus	aIPS	L	279	48.3	58.6	34.1	4mo ​< ​1yo
	Middle Frontal Gyrus	DLPFC	R	262	−24.9	−38.1	30.1	4mo ​> ​1yo
	Middle Temporal Gyrus	VOC	L	180	51	58.5	9.5	
	Superior Frontal Gyrus		L	110	17.5	−38.9	35.8	
**Age x Hb** 1yo v 2yo	Inferior Parietal Lobule	aIPS	R	163	−48.1	41.9	49.2	1 yo ​> ​2yo
**SS x Hb** SS med v SS high	Angular Gyrus	aIPS	L	124	46.1	55.6	32.8	SS med ​> ​SS high

**Note:** Clusters were localized using the center of mass xyz coordinates and labels were derived from the MNI atlas (Eickhoff-Zilles ​macro ​labels ​from ​N27 in AFNI). The ROI column indicates that a portion of the cluster was overlapping or near a target ROI.

**Fig. 3:**
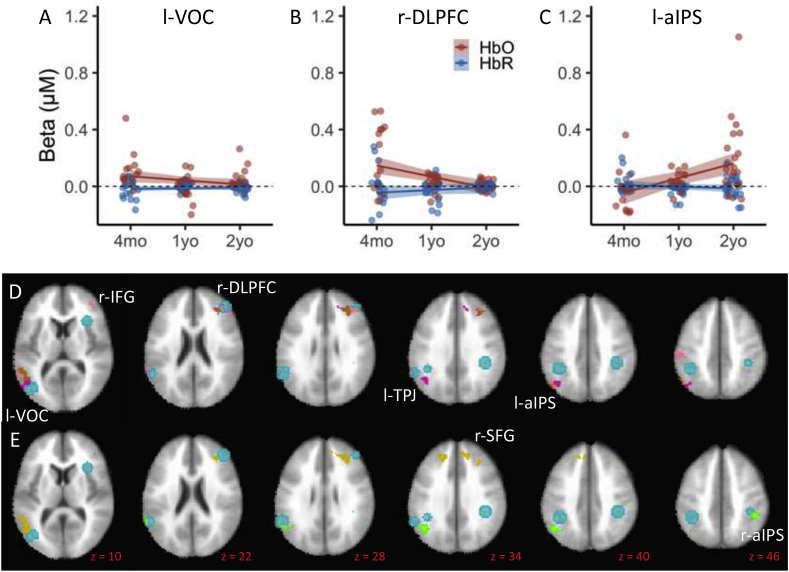
fNIRS ANOVA and linear contrast results. The line plots on the top panels show how the VWM network changed across ages in early development. Red lines/dots show HbO, blue lines/dots show HbR, shading depicts standard error. Panels show patterns of functional brain activity as a function of age in the left Ventral Occipital Cortex (VOC, A), the right Dorsolateral Prefrontal Cortex (DLPFC, B), and the left anterior Intraparietal Sulcus (aIPS, C). Brain images show significant clusters from the fNIRS ANOVA after familywise correction. Row D shows Hb and Age x Hb ANOVA results: pink ​= ​chromophore (Hb) effects, fuschia ​= ​Age x Hb effects, and brown ​= ​overlap between Hb and AgexHb effects. Row E shows Age x Hb general linear tests: mustard ​= ​4mo ​> ​1yo, and light green ​= ​1yo ​> ​4mo. ROIs from the adult fMRI literature are shown as teal circles.

Fig. 3D shows the Hb and Age x Hb effects from the ANOVA, while Fig. 3E shows the significant effects from the Age linear contrasts. There was considerable overlap between these significant fNIRS clusters and the VWM network identified in fMRI studies with adults (see teal ROI circles in Fig. 3D and E): fNIRS clusters overlapped or were near 6 of 19 target ROIs (see ‘ROI’ column in Table 1, Table 2). In particular, there was robust neural activation near r-IFG, r-DLPFC extending up into SFG, left ventral occipital complex (l-VOC), l-TPJ, and bilateral aIPS. Thus, in contrast to hypothesis 1 that VWM in early development is not localized in fronto-parietal cortex, we found task-specific functional activation in the canonical VWM network in the outer cortex. This is consistent with hypothesis 4.

All of the Hb effects shown in Fig. 3D had greater concentrations of HbO than HbR; thus, the chromophore effects showed a canonical pattern. The three significant Age x Hb effects are shown in Fig. 3A–C. There was a decrease in activation over ages in l-VOC and r-DLPFC, and an increase in activation over age in l-aIPS. In all cases, we found a canonical chromophore effect with HbO ​> ​HbR. The Age linear contrasts shown in Fig. 3E help clarify these effects. Notably, the Age x Hb contrasts were only significant when comparing 4-month-olds and 1-year-olds. In particular, there was greater activation in l-VOC, r-DLPFC, and bilateral SFG for 4-month-olds, and greater activation in bilateral aIPS for 1-year-olds. Given that there were no significant clusters with greater activation for 2-year-olds relative to 1-year-olds (and, therefore, no clusters where the conjunction of contrasts was significant), there were not strong linear Age trends in the data; rather, age-related differences were primarily focused in the first year with a plateau (or non-significant increase) in the pattern of activation thereafter.

**Fig. 4:**
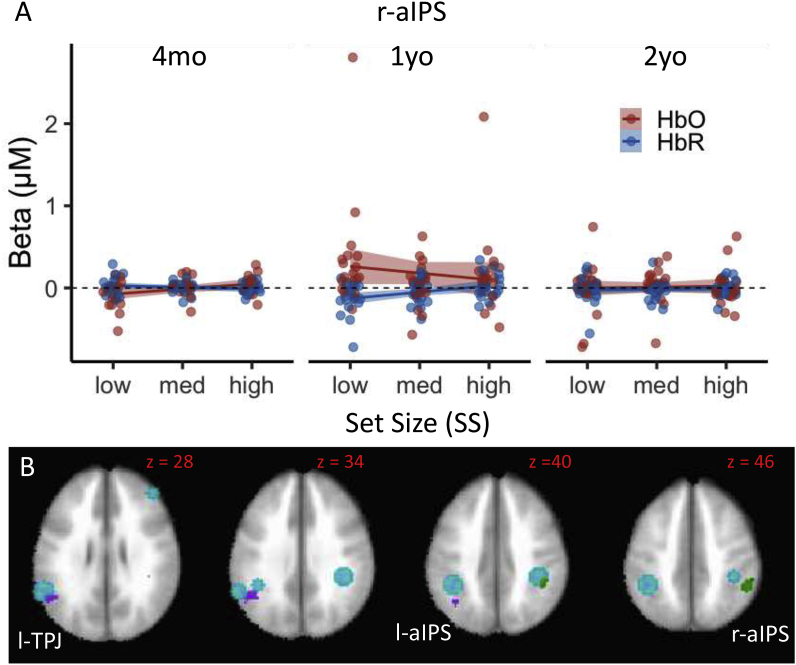
SS-related effects from the ANOVA and linear contrasts. The line plots in panel A shows patters of brain activity in right anterior Intraparietal Sulcus (aIPS) as a function of memory load (set size). Red lines/dots show HbO, blue lines/dots show HbR, shading depicts standard error. Panel B shows the Age x SS x Hb effect from the ANOVA: dark green ​= ​Age x SS x Hb effect; purple shows the significant cluster from the SS linear contrasts with SS med ​> ​SS high. ROIs from the adult fMRI literature are shown as teal circles.

Fig. 4 shows the SS-related effects from the ANOVA and linear contrasts. All SS-related effects were centered near bilateral aIPS and l-TPJ. The r-aIPS cluster in Fig. 4B shows the Age x SS x Hb effect from the ANOVA. As can be seen in Fig. 4A, there was a decrease in activation as the set size increased for 1-year-olds. This is consistent with the Age contrasts shown in Fig. 3E which indicated that activation in r-aIPS was greater for 1-year-olds relative to 4-month-olds. The SS linear contrasts revealed one cluster near l-aIPS and extending ventrally into l-TPJ where activation at SS2 was greater than activation at SS3 (see Fig. 4B). Note that the absence of any significant clusters in the SS1 vs SS2 contrasts indicates that there were not strong linear trends over SS; rather, activation at SS1 and SS2 appeared comparable with a decrease in activation at the highest SS.

*Brain-behavior relationships*. To better understand the functional roles of each significant cluster of task-related brain activity from the ANOVA, we ran linear models examining whether individual differences in change preference scores and visual exploratory measures (total looking time/shift rate) predicted brain activity (see Table 3). Note that total looking time and shift rate measures are inversely correlated, such that infants showing low total looking times typically have high shift rates (and vice versa). This is consistent with models of visual exploration in early development where high shift rates have been used as a marker of fast visual information processing which ultimately leads infants to look away from the task display (i.e., low total looking, see Perone et al., 2011; Perone and Spencer, 2013).

**Table 3:** Significant brain-behavior relationships.

Cluster	ROI	Behavioural Measure	Omnibus F	Omnibus p	Effect	t	p assoc. with t
Middle Temporal Gyrus	l-VOC	CP	2.505	0.001	CP∗Hb	4.672	0.032
					CP∗SS	9.313	<0.001
					CP∗Age∗SS	2.664	0.033
					CP∗SS∗Hb	6.859	0.001
Superior Temporal Gyrus	l-TPJ	CP	1.965	0.005	CP∗Age	3.108	0.046
					CP∗Hb	6.917	0.009
					CP∗SS	3.886	0.022
					CP∗SS∗Hb	5.312	0.005
		TL	1.834	0.008	TL∗Age	3.070	0.048
Middle Frontal Gyrus	r-DLPFC	TL	2.317	0.001	TL	10.737	0.001
					TL∗Age	3.645	0.027
					TL∗Hb	5.502	0.020
Angular Gyrus	l-alPS	SR	1.618	0.028	SR∗Age	3.176	0.043
Inferior Parietal Lobule	r-alPS	SR	1.932	0.005	SR∗Age	3.863	0.022

*Note:* Omnivous p values are corrected; p values associated with t are uncorrected.

**Fig. 5:**
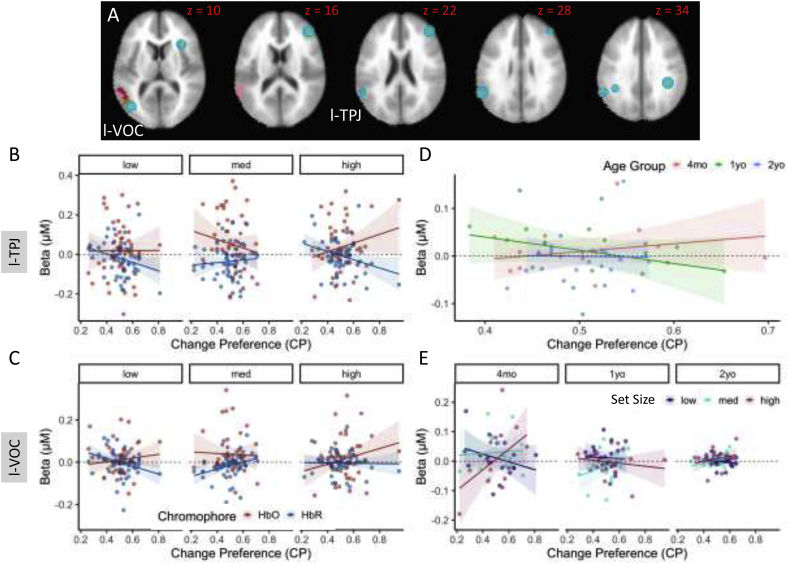
Relationships between change preference scores and functional brain activity. Panel A shows clusters in left VOC and left TPJ whose activity was significantly predicted by change preference scores. The line plots in the bottom panels show results from models predicting neural activity with behavior. Panel B shows the CP∗SS∗Hb interaction from l-TPJ (see Table 3), while panel C shows the same effect from l-VOC. Panel D shows the significant CP∗Age interaction in l-TPJ, while panel E shows the CP∗Age∗SS interaction in l-VOC. Colors are indicated by the legends. Lines and dots follow the same color scheme. In all line plots, shading depicts standard error.

Fig. 5 shows that change preference (CP) scores significantly predicted brain activity in l-TPJ (see Table 3), consistent with the SS effects observed in l-TPJ reported above – infants with higher CP scores showed greater activation in this brain region in the low and high load conditions (Fig. 5B). A similar pattern was evident in l-VOC (Fig. 5C). We conducted follow-up tests in both regions, splitting by SS. These tests revealed a robust CP ​× ​Hb interaction in both the low and high loads, but not in the medium load condition.

TPJ and VOC also showed interactions between CP and Age. In particular, there was a CP ​× ​Age interaction in l-TPJ such that 4-month-olds with higher CP scores showed greater activation in this brain region, while 1-year-olds with higher CP scores showed suppression in TPJ (Fig. 5D). The suppression of l-TPJ activation with better VWM performance is consistent with fMRI studies with adults which report negative BOLD in l-TPJ as the WM load is increased (Todd et al., 2005). The pattern of effects in l-VOC was generally similar but showed an interaction with Load. In particular, 4-month-olds with higher CP scores showed greater activation in the high load condition, with suppression in the low load condition (Fig. 5E). One-year-olds with higher CP scores, by contrast, generally showed suppression in l-VOC, consistent with the pattern in l-TPJ.

**Fig. 6:**
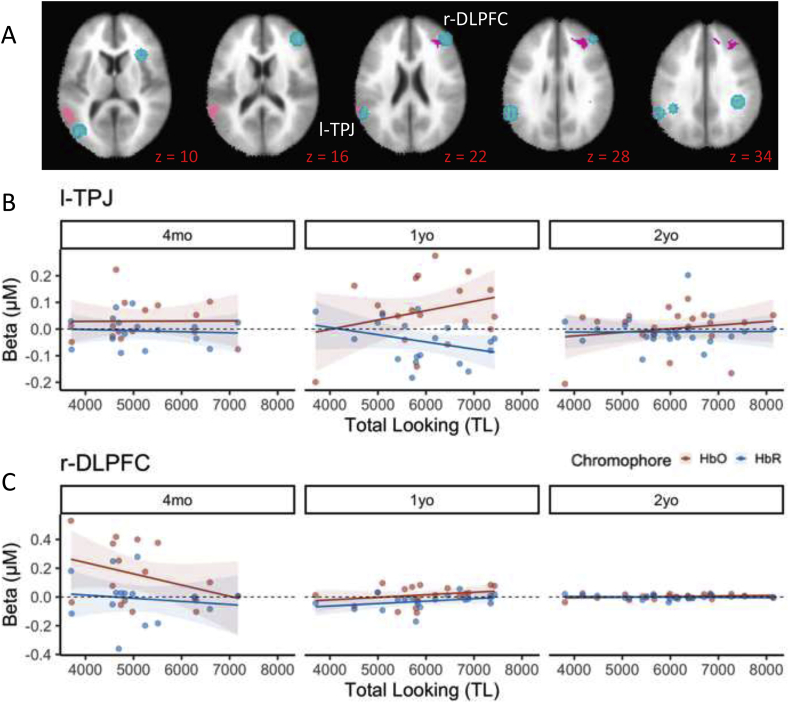
Relationships between brain activity and total looking time. Panel A shows clusters in left TPJ and right DLPFC whose activity was significantly predicted by total looking time. Panel B shows the TL∗Age interaction from l-TPJ (see Table 3) plotted for each chromophore separately for consistency with panel C. Panel C shows the TL∗Age effects from r-DLPFC, plotted separately for each chromophore to highlight the TL∗Hb effect in this region. Colors are indicated by the legend. Shading depicts standard error.

Fig. 6 shows that two brain regions – l-TPJ and r-DLPFC – showed relationships between individual differences in total looking time and brain activity. In particular, l-TPJ showed a TL x Age effect, while r-DLPFC showed effects of TL, TL x Age, and TL x Hb. Fig. 6 shows the TL x Age effects for each cluster in the context of the chromophore effect for consistency with previous figures. In r-DLPFC, faster-processing 4-month-olds (low TL) showed greater activation, while slower-processing 1-year-olds showed greater activation in l-TPJ. Thus, as with CP scores, there was once again a developmental flip in the pattern of activation between 4 months and 1 year of age. Note that the pattern of results across the CP and TL analyses is consistent with prior reports suggesting that higher CP scores are associated with faster visual processing (e.g., higher shift rates and lower total looking; see Simmering, 2016).

**Fig. 7:**
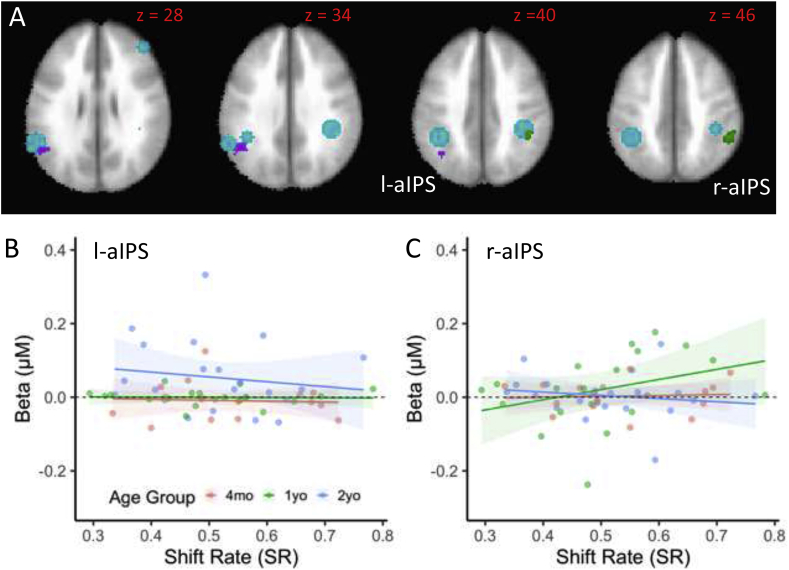
Relationships between brain activity and shift rate. Panel A shows l-aIPS and r-aIPS clusters showing a significant relationship to shift rate over ages. Panels B (l-aIPS) and C (r-aIPS) show significant Shift Rate ​× ​Age interaction in linear models predicting brain activity from behavioral measures (see Table 3). Colors are indicated by the legend.

The final brain-behavior relationships are shown in Fig. 7. Faster-processing 1-year-olds with a higher shift rate showed greater activation in r-aIPS (Fig. 7C). The high activation for 1-year-olds in this region is consistent with the ANOVA results shown in Fig. 4A. By contrast, slower-processing 2-year-olds with a lower shift rate showed greater activation in l-aIPS (see Fig. 7B). Considered together, these data suggest a developmental refinement in the role aIPS plays in shifts of attention and change detection between 1 and 2 years.

## 4 Discussion

The goal of this study was to use image-based fNIRS to probe the spatial organization of the VWM network in early development, testing four functional localization hypotheses. Results failed to support hypothesis 1 that VWM in infancy is not localized within a fronto-parietal network; rather, we found localized task-specific activation near 6 of 19 ROIs in cortex. We cannot rule out the involvement of the medial temporal lobe in VWM in infancy due to the limitations of fNIRS. Nevertheless, our data show that core parts of the cortical VWM network are engaged very early in development.

Notably, engagement of the VWM network was not isolated to posterior cortex as suggested by hypothesis 2. Rather, we found task-specific localized activation in large portions of frontal cortex including DLPFC—a hub for working memory in previous work (Buss et al., 2014; Edin et al., 2009). We also found significant task-related activation in r-IFG and SFG. Thus, the VWM network appears to be engaged in a system-wide manner that includes both frontal and posterior cortices. Note that r-SFG is a key site in the frontal attention network (Petersen and Posner, 2012). The involvement of SFG here may reflect our use of a preferential looking task to test VWM which places heavy demands on shifts of visual attention.

The third hypothesis we tested focused on the laterality of the VWM network. Our ANOVA results showed robust activation in both hemispheres; however, brain-behavior correlations showed evidence of functional laterality. The clusters showing the only association with CP scores were in the left hemisphere (Fig. 5). This is consistent with Kwon et al. (2002) who reported a left-lateralized network for WM-related rehearsal; however, Kwon et al. localized this network to VLPFC, while our findings were localized in the posterior cortex (TPJ, VOC). Our findings were less consistent with evidence from Kwon et al. regarding a right-lateralized visual attention network. In particular, two associations with visual exploratory measures were right lateralized (r-DLPFC, r-aIPS), while two were left lateralized (l-TPJ, l-aIPS).

The final hypothesis we considered was based on recent evidence of an adult-like spatial organization for faces and scenes by 4 months of age (Deen et al., 2017). In some respects, our data are consistent with this finding in that we found strong activation in l-SFG, r-DLPFC, l-VOC, and l-TPJ by 4 months. Thus, aspects of the VWM network appear to become functional relatively early in the first year. Notably, r-DLPFC showed an early association with total looking time. This suggests that one of the first achievements in infancy is to regulate and control looking—looking back and forth between displays, controlling consolidation in VWM, and regulating the release from fixation (Perone et al., 2011; Perone and Spencer, 2013).

Although aspects of VWM functional activation are evident by 4 months, our data also show considerable change between 4 months and 1 year consistent with behavioral results from Ross-Sheehy et al. (2003). Most of the developmental changes at 1 year were focused near bilateral aIPS. Several studies with adults have proposed that aIPS is the likely site of VWM (Todd et al., 2005; Todd and Marois, 2005, 2004). For instance, activation in aIPS is modulated by VWM capacity and shows an increase in activation as the memory load is increased with a plateau at supra-capacity set sizes. Consistent with these data, all of our SS-related effects were localized to bilateral aIPS. Critically, however, there appears to be a developmental difference in that activation *decreases* at high set sizes. This replicates data from Buss et al. (2014) where we found a decrease in right parietal activation at the highest set sizes as 3 and 4-year-olds completed a change detection task. Interestingly, we found a similar decrease in activation at high memory loads in aging adults as well (Wijeakumar et al., 2017b). Considered together, these data suggest that the plateau in parietal activation at supra-capacity set sizes is a developmental achievement that emerges sometime during childhood. Interestingly, we did not see large differences in brain activity between 1 and 2 years, although data from r-aIPS showed a quantitative increase in activation at 2 years (see Figure 3C) and 2-year-olds with a lower shift rate showed greater l-aIPS activation than the other age groups. These findings suggest that there is some refinement in VWM processes centered on aIPS between 1 and 2 years.

In addition to developmental changes in aIPS, we found developmental differences in l-TPJ and l-VOC activation. These regions showed robust relationships with change preference scores—a key index of VWM in early development (Oakes et al., 2011; Oakes et al., 2006; Ross-Sheehy et al., 2003). Interestingly, we found a developmental flip in activation such that 4-month-olds with higher CP scores show greater activation while 1-year-olds with higher CP scores showed greater suppression. l-TPJ has been implicated in VWM in previous work (Buss et al., 2014; Todd and Marois, 2004, Todd and Marois, 2005) and shows an increasingly negative BOLD signal as the memory load is increased with adult participants (Todd et al., 2005). It is possible the developmental flip in our data reflects the emergence of distractor suppression (see Suzuki and Gottlieb, 2013) in this brain region by 1 year of age. This may be critical in the preferential looking task as both displays contain blinking, colored squares; thus, infants must suppress looking to, for instance, the non-changing display as they consolidate the items on the changing display. It is notable that l-TPJ was the only region associated with both CP scores *and* visual exploratory scores, suggesting that this is a hub region for VWM in early development.

Considered together, our findings support the utility of fNIRS image-reconstruction in early development, consistent with previous validation studies (Ferradal et al., 2014; Wijeakumar et al., 2017a; Wijeakumar et al., 2015). Although our data reveal considerable overlap with the VWM network identified in fMRI studies with adults, not all patterns of activation were precisely localized. For instance, although we found a significant cluster of activation near r-IFG (see Fig. 4D), this cluster did not overlap with ROIs from the adult fMRI literature (see Wijeakumar et al., 2015). It is possible that this reflects limitations in image reconstruction caused by our use of age-specific MRI atlases instead of individual-specific brain anatomy. This could be addressed in future work that combines structural MRI with image-based fNIRS. Another possible limitation of fNIRS is its sensitivity to physiological contamination. We reduced the impact of such influences by using an event-related design that de-synchronized the task events from physiological cycles such as heart rate and respiration. We also combined information from both chromophores by including this as a factor in the analysis and used short source-detector distances to regress out physiological signals. As with any new neuroimaging technique, it will be important in future work to further validate image-reconstructed fNIRS approaches. Until such work is completed, we need to interpret findings with caution.

In summary, our findings reveal—for the first time—that the functional VWM network shows robust engagement of similar brain regions identified in fMRI studies with adults as early as four months with subsequent refinement of visual exploratory and VWM-related processes by 1 year of age. In this sense, there is developmental consistency in the spatial localization of effects consistent with hypothesis 4. In addition, our data were generally consistent with a proposed left lateralized VWM network consistent with hypothesis 2. Finally, our findings showed the emergence of robust activation in bilateral aIPS, l-TPJ, and l-VOC at 1 year of age as VWM improves, highlighting the importance of these brain regions in VWM consistent with previous fMRI and fNIRS work (Buss et al., 2014; Todd and Marois, 2004, Todd and Marois, 2005).

These results raise key questions for future work. One issue is to understand the developmental cascade that drives the functional organization of the VWM network prior to four months. Image-based fNIRS might play a key role in exploring this question as this technology can be used with very young infants (Ferradal et al., 2016). It is also critical to extend this work to longitudinal studies to examine whether the developmental changes in functional organization reported here are stable within individuals. In particular, do we see, for instance, that early l-VOC/r-DLPFC/r-SFG activation is followed by later bilateral aIPS/l-TPJ activity *within individuals*? If so, are such patterns predictive of individual differences in VWM outcomes? Such a result could be useful as a biomarker within individuals to assess risk early in development and to monitor changes in the functional organization of the VWM network to help guide interventions.

## Author contributions

LDR, SW, and JPS designed the study. LDR and SW supervised data collection. All authors contributed to data analysis, including image reconstruction analyses. LDR and JPS wrote the manuscript. All authors commented on the final version.

## CRediT authorship contribution statement

**Lourdes Delgado Reyes:** Conceptualization, Methodology, Software, Formal analysis, Investigation, Data curation, Writing - original draft, Writing - review & editing, Visualization, Project administration. **Sobanawartiny Wijeakumar:** Conceptualization, Methodology, Software, Formal analysis, Investigation, Writing - review & editing, Project administration. **Vincent A. Magnotta:** Software, Formal analysis, Writing - review & editing. **Samuel H. Forbes:** Formal analysis, Visualization, Writing - review & editing. **John P. Spencer:** Conceptualization, Methodology, Formal analysis, Resources, Writing - original draft, Writing - review & editing, Visualization, Supervision, Funding acquisition.

## Declaration of competing interest

We declare no competing interests.

## Footnotes

1 We use the term ‘infancy’ to refer to the period from birth to 2 years of age and ‘infants’ to refer to children whose age falls within this range.
